# Correlative in-resin super-resolution and electron microscopy using standard fluorescent proteins

**DOI:** 10.1038/srep09583

**Published:** 2015-03-31

**Authors:** Errin Johnson, Elena Seiradake, E. Yvonne Jones, Ilan Davis, Kay Grünewald, Rainer Kaufmann

**Affiliations:** 1 Sir William Dunn School of Pathology, University of Oxford, South Parks Road, Oxford, OX1 3RE, UK; 2 Division of Structural Biology, Wellcome Trust Centre for Human Genetics, University of Oxford, Roosevelt Drive, Oxford, OX3 7BN, UK; 3 Department of Biochemistry, University of Oxford, South Parks Road, Oxford, OX1 3QU, UK

## Abstract

We introduce a method for correlative in-resin super-resolution fluorescence and electron microscopy (EM) of biological structures in mammalian culture cells. Cryo-fixed resin embedded samples offer superior structural preservation, performing in-resin super-resolution, however, remains a challenge. We identified key aspects of the sample preparation procedure of high pressure freezing, freeze substitution and resin embedding that are critical for preserving fluorescence and photo-switching of standard fluorescent proteins, such as mGFP, mVenus and mRuby2. This enabled us to combine single molecule localization microscopy with transmission electron microscopy imaging of standard fluorescent proteins in cryo-fixed resin embedded cells. We achieved a structural resolution of 40–50 nm (~17 nm average single molecule localization accuracy) in the fluorescence images without the use of chemical fixation or special fluorophores. Using this approach enabled the correlation of fluorescently labeled structures to the ultrastructure in the same cell at the nanometer level and superior structural preservation.

Correlative light and electron microscopy (CLEM) techniques, which allow fluorescently labeled molecules of interest to be placed in their correct ultrastructural context within the same cell, are now becoming widely used in various fields of biological research^1^. However, there is a substantial gap between the resolution of fluorescence microscopy (several hundred nanometers) and the sub-nanometer resolution of electron microscopy (EM), which often impedes the correlation quality and complicates interpretation of the data. Various super-resolution methods^2^,^3^,^4^,^5^,^6^,^7^ have been developed in the field of fluorescence microscopy over the last 20 years to overcome the diffraction-limited resolution in light microscopy. These techniques are now being combined with EM to correlate ultrastructure and the corresponding distribution of fluorescent markers with a much higher degree of precision and detail compared to conventional CLEM^5^,^8^,^9^,^10^.

To date, the highest resolution achieved with super-resolution fluorescence microscopy has been in chemically fixed samples, as live-cell super-resolution microscopy remains technically challenging^11^,^12^. Therefore, the majority of super-resolution CLEM results have so far been attained with photoactivated localization microscopy (PALM), direct stochastic optical reconstruction microscopy (dSTORM) and stimulated emission depletion (STED) microscopy on chemically fixed samples, either using genetically encoded photo-activatable molecules or antibody labeling after fixation^5^,^9^,^10^. As chemical fixation is associated with structural changes in the sample^13^,^14^ an alternative, albeit more technically challenging approach, is to fast freeze the living sample to preserve it in a near-native state (vitrification).

Indeed, first proof of principle experiments have shown the feasibility of super-resolution fluorescence cryo-microscopy^15^ and correlation with cryo-EM^16^, but the resolution achieved to date is below that at ambient temperatures due to the technical challenges of fluorescence cryo-microscopy setups, particularly the current lack of dedicated cryo-immersion objectives which severely limits the obtainable numerical aperture^15^,^17^. Vitrified samples can instead be freeze substituted and embedded in resin at low temperature, which combines the enhanced structural preservation of fast freezing techniques with the advantages of imaging with high numerical aperture lenses at ambient temperatures^18^. Furthermore, imaging the sample in resin sections with fluorescence microscopy followed by EM is advantageous, as EM sample processing can induce substantial structural and spatial changes in the sample^9^,^19^ that affect the quality of correlation, especially at the level of resolution achieved with super-resolution fluorescence microscopy.

Vitrification of living samples followed by freeze substitution is incompatible with the more commonly used methods of immunolabeling proteins of interest using fluorescent antibodies as this generally requires permeabilizing cells to permit reagent entry, which damages cell membranes at the ultrastructural level^13^. Alternatively, the EM preparation procedure may be modified to preserve antigenicity so that proteins can be immunolabeled for post-embedding CLEM on resin sections^20^, though drawbacks of this technique include mis-localization of antigens and/or a significant reduction in labeling efficiency^21^. The Tokuyasu technique^22^ allows sectioning without resin embedding of the sample, which makes it more accessible for immunolabeling^23^, but this method involves chemical fixation and cryo-sectioning, which is technically challenging. However, the general disadvantage of immunofluorescence-based approaches with regards to super-resolution CLEM is that immunolabeling can require extensive optimization to obtain a specific signal with minimal background fluorescence^13^,^24^, which is a critical factor for super-resolution imaging. Therefore, it is desirable to instead use genetically encoded fluorescent fusion proteins expressed in living cells to label proteins of interest. Protein localization with nanometer accuracy has been reported for Citrine and tdEos molecules in resin sections after freeze substitution using STED and PALM^8^. However, the reported structural resolution was less than what has been achieved with similar fluorophores in chemically fixed samples or living cells^25^,^26^, as well as in cryosections with the Tokuyasu technique^27^, indicating that the fluorescence and/or photo-switching properties of these proteins are affected by the EM sample preparation procedure. High quality imaging of fluorescent proteins (FPs) in resin sections therefore requires the use of a freeze substitution protocol that preserves both fluorescence and ultrastructure, while introducing sufficient contrast for subsequent transmission electron microscopy (TEM) imaging. For basic wide-field and confocal CLEM, this approach has been successfully employed with in-resin GFP and YFP fluorescence^19^,^28^,^29^,^30^. However, most super-resolution microscopy techniques are further based on switching of the fluorescent molecules^31^ to increase the resolution beyond the diffraction limit of light^31^. For in-resin super-resolution correlative imaging, the challenge is therefore to maintain not only the fluorescence itself throughout the sample preparation procedure, but also the switching capabilities of the FPs.

Here, we describe a novel method for freeze substitution and resin embedding of mammalian culture cells to maintain the photo-switching capabilities of genetically encoded standard fluorescent proteins, such as GFP. This enabled us to use a single molecule localization microscopy (SMLM) technique that has been developed for standard fluorescent proteins^32^,^33^,^34^ and apply it for the first time to ultrathin resin sections, reaching a structural resolution of ~50 nm in fluorescence microscopy. Overlaying these super-resolution images with that of the EM ultrastructure in the same cell allowed us to correlate structural features with an unprecedented level of subcellular detail without the use of chemical fixation or specialized fluorescent proteins.

## Results

### Adaptation of high pressure freezing and freeze substitution to enable in-resin SMLM

**Figure 1:**
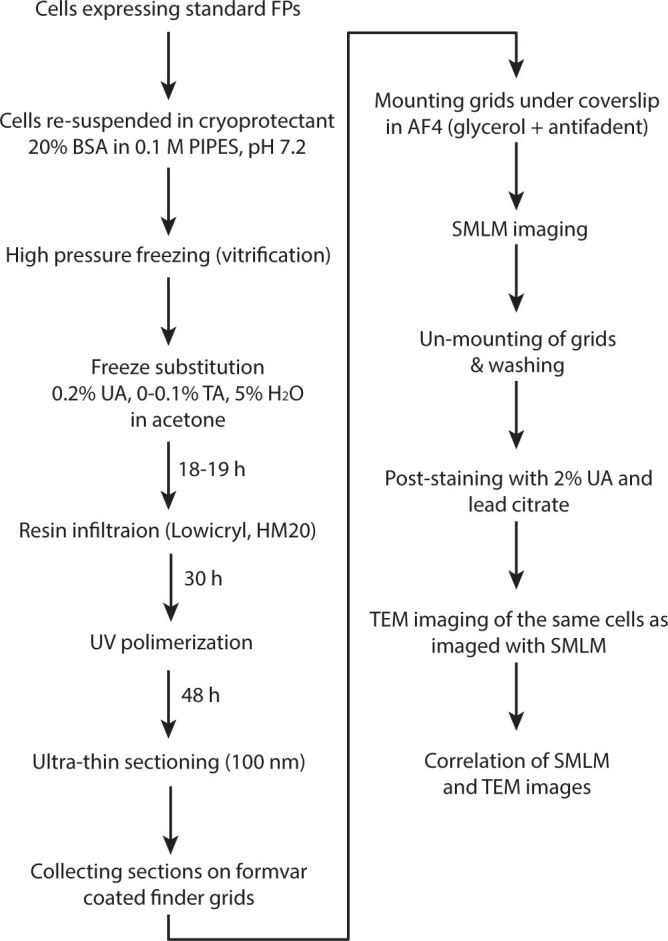
Overview of sample preparation steps for correlative in-resin super-resolution and TEM imaging. UA: uranyl acetate, TA: tannic acid.

The most critical parameters for successful correlative in-resin SMLM and EM imaging are the choice of cryoprotectant, freeze substitution medium and freeze substitution length. The challenge was to find the optimal balance between high quality freezing, sufficient EM contrast, preservation of in-resin fluorescence and, importantly, preservation of the photo-switching capabilities of standard FPs in mammalian culture cells. An overview of the optimized workflow is shown in Fig. 1.

The use of 20% BSA in 0.1 M PIPES buffer pH 7.2 as the cryoprotectant produced minimal background autofluorescence, a problem that was encountered when the cells were frozen directly in the growth medium and which can severely reduce the quality of super-resolution imaging. It also resulted in well frozen cell pellets that were more robust during handling than those frozen re-suspended in 10% BSA prior to freezing.

**Figure 2:**
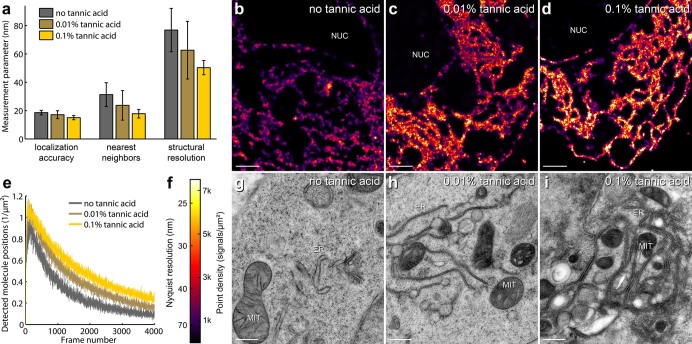
The quality of SMLM imaging is dependent on addition of tannic acid to the freeze substitution medium. (a) Comparison of average single molecule localization accuracy, nearest neighbor distances and structural resolution of EphA2-mVenus localized in the plasma membrane and the endoplasmic reticulum (ER) of HEK293T cells in resin sections for samples with different concentrations of tannic acid in the freeze substitution medium. Nearest neighbor distances have been determined considering the 20 nearest molecule positions around each position. Error bars represent standard deviations. (b–d) Example SMLM images of cells, all acquired under the same conditions, depicting typical results for different concentrations of tannic acid. Scale bars are 1 μm. (e) Detected single molecule signals per μm^2^ and image frame during SMLM data acquisition for different concentrations of tannic acid in the freeze substitution medium. Only areas of fluorescently labeled structures were included. Each curve represents the average values of the SMLM measurements (total 57) for each concentration of tannic acid. (f) Color code for local density of detected molecules and Nyquist limited resolution in SMLM images (b–d). (g–i) Representative TEM images showing the typical quality of the ultrastructure (ER and mitochondria (MIT)) for freeze substitution with different concentrations of tannic acid. Scale bars are 0.5 μm. Note that the TEM images in (g–i) depict different cells than those shown in the SMLM images (b–d).

We found that the composition of the freeze substitution medium was the most crucial parameter for the preservation of both fluorescence and photo-switching capabilities of FPs. The best contrast agent for EM is osmium tetroxide, which is a strong oxidizer and quenches fluorescence of standard fluorescent proteins^35^ making it generally unsuitable for CLEM techniques involving in-resin fluorescence. Instead, 0.1–0.2% uranyl acetate has been used to enhance EM contrast without affecting fluorescence^19^,^28^,^36^ and here we found that at these concentrations uranyl acetate also did not negatively impact FP photo-switching for SMLM imaging. However, because uranyl acetate is not as strong a contrast agent for EM as osmium tetroxide, we investigated whether different concentrations of tannic acid, which has been employed during freeze substitution to enhance membrane staining^37^, could be used to further augment contrast for EM imaging without impeding in-resin fluorescence. Instead, we found that the addition of tannic acid to the freeze substitution medium greatly enhanced the quality of SMLM imaging (Fig. 2). The use of tannic acid improved both the single molecule localization accuracy *σ* (due to an increased signal to noise ratio) and the photo-switching of the FPs, resulting in a higher local density *ρ* of detected single molecule positions (Fig. 2a). Both parameters are critical for the achievable structural resolution (2D): 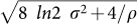
38. The addition of tannic acid to the freeze substitution medium also had an effect on the photo-switching kinetics of standard FPs. A lower concentration of tannic acid resulted on average in a lower rate of FPs recovering to the fluorescent state (Fig. 2e). The impact of different tannic acid concentration on the photo-switching of standard FPs is also qualitatively illustrated in the corresponding SMLM images in Fig. 2b–d. Lowering the tannic acid concentration from 0.1% to 0.01% resulted in reduced and more variable SMLM image quality (Fig. 2a–d). Conversely, the quality of ultrastructural preservation in samples freeze-substituted with 0.1% tannic acid (Fig. 2i) was slightly impaired in comparison to those treated with less or no tannic acid (Fig. 2g, h). Indeed, it has previously been reported that 1% tannic acid in the freeze substitution medium can lead to structural changes in the sample^39^, and as such we did not use more than 0.1% tannic acid in the freeze substitution medium. The addition of 5% water to the freeze substitution medium slightly increased the in-resin fluorescence, as described previously^19^.

Peddie et al.^19^ reported that a short freeze substitution protocol was required for strong in-resin fluorescence in mammalian cells. We compared three different freeze substitution protocols: a long freeze substitution (60–80 h), according to Kukulski et al.^28^; quick freeze substitution (QFS, 3 h), according to Peddie et al.^19^ and intermediate freeze substitution (IFS, 20 h), modified from Hawes et al.^40^. The long freeze substitution protocol resulted in weaker in-resin fluorescence, but drastically reduced photo-switching of FPs (Supplementary Fig. 1). The QFS and IFS protocols both strongly preserved the fluorescence of standard FPs and their photo-switching capabilities. The IFS protocol runs overnight, which enabled longer resin infiltration times to be used compared to the QFS infiltration procedure^19^, where the resin infiltration was performed the same day as the freezing and QFS. In our hands, the longer infiltration times of the IFS resulted in more consistently well-polymerized blocks. The cryo-preparation and resin embedding procedure did not alter the distribution of FP fluorescence (in this example EphA2-mVenus), as the observed signal was comparable to what has previously been reported in live HEK293T cells^41^.

### Sample mounting for in-resin SMLM imaging

The critical points to consider when mounting samples for correlative SMLM and EM imaging are (i) use of a mounting medium that facilitates single molecule photo-switching of standard FPs, (ii) possibility of un-mounting without losing the sections from the grid, and (iii) use of a mounting medium that does not produce precipitates in the subsequent TEM imaging and can easily be washed off. Resin sections on finder grids were mounted between a microscope slide and a glass coverslip, with the sections facing the coverslip, using a glycerol based mounting medium with antifadent (AF4, Citifluor) and sealed with nail polish. Sections mounted in glycerol based media were less prone to adhere to the coverslip after un-mounting and also showed an increased photo-switching quality. We found that formvar film on the finder grids increased the adhesion of the sections, protecting them during the un-mounting process, and also increased section stability under the electron beam.

### In-resin SMLM imaging

Using our SMLM-adapted freeze substitution and infiltration protocol, we achieved a structural resolution in the range of 40–50 nm using SMLM with standard FPs (GFP, mGFP, mVenus, mRuby2) in resin sections of mammalian cells. Here, SMLM was performed under similar conditions to those described in earlier work for super-resolution localization microscopy using standard FPs in chemically fixed samples^32^,^33^,^34^. Determination of fluorescent molecule positions and visualization of the data was performed as described previously^42^,^43^. The number of photons detected for the single molecule signals was in the range of 100–1000. The ‘on-time’ of the fluorophores was in the range of ~60 ms (depending on laser intensity), with the lifetime of the dark state in the range of many seconds up to minutes. The single molecule localization accuracies we obtained were on average 14–20 nm (depending on the particular FP type). These values are similar to what has been reported for non-embedded samples using standard FPs^32^,^33^,^34^. The crucial parameter for SMLM imaging is the structural resolution. To assess this, we determined the local point densities and nearest neighbor distances in the SMLM data sets to estimate the Nyquist limited resolution. This was in the range of 20–50 nm, with some variation between cells due to differences in expression level of the FPs.

### Correlative in-resin super-resolution and electron microscopy

**Figure 3:**
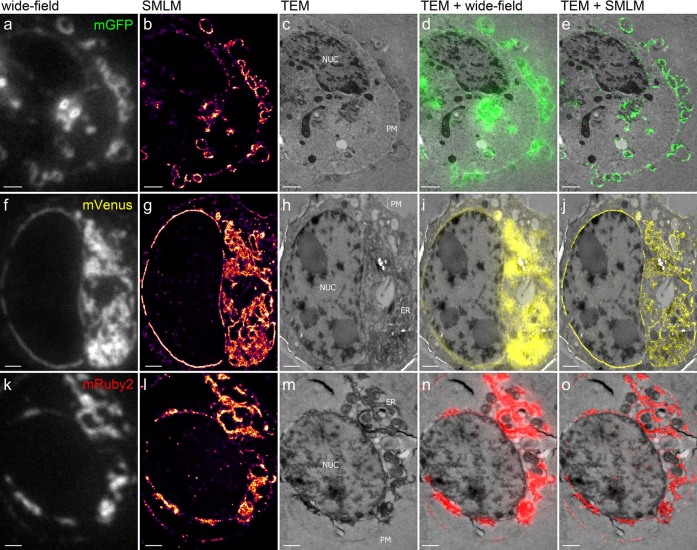
Correlative in-resin super-resolution fluorescence and EM imaging of HEK293T cells transfected with EphA2/A4 receptor proteins fused to mGFP, mVenus or mRuby2. The first column shows conventional wide-field fluorescence images, the second column the corresponding SMLM images with color coded local densities of detected molecules and the corresponding Nyquist limited resolution (the same color code as in Fig. 2f and Fig. 4f applies). TEM images of the same cells are depicted in the third column (plasma membrane (PM), ER and the nucleus (NUC) are indicated in the individual images), followed by an overlay of the TEM images with the conventional wide-field fluorescence images, and an overlay of TEM and SMLM images. Scale bars are 1 μm. The freeze substitution for these cells was performed with 0.1% TA.

**Figure 4:**
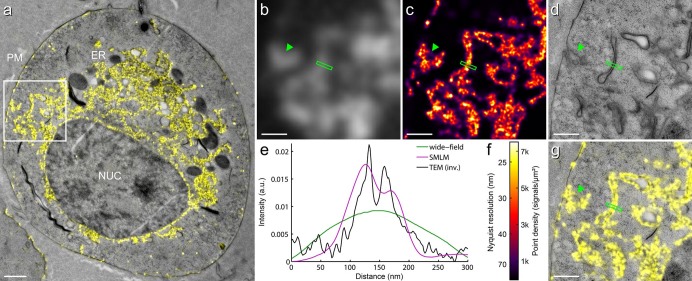
Comparison of resolution achieved with in-resin super-resolution CLEM. (a) Overlay of SMLM image on the TEM image of EphA2-mVenus in a resin embedded HEK293T cell. Plasma membrane (PM), ER and the nucleus (NUC) of the cell are indicated in the image. The rectangle marks the region shown with a higher magnification in the panels on the right: (b) Conventional wide-field fluorescence microscopy, (c) Super-resolution SMLM and (d) TEM. The line profiles in (e) show the different levels of details of membrane structures resolvable with each technique. The structural resolution of ~50 nm achieved with SMLM in resin sections using standard fluorescence proteins (here mVenus) allowed a superior correlation of fluorescent signals and EM ultrastructure (a, g). Scale bar is 1 μm in (a) and 0.5 μm in (b, c, d, g). Arrow heads point to sites of endocytosis of EphA2-mVenus from the plasma membrane into vesicles. Note that the line profile for the TEM image was generated from inverted pixel values for better comparison. (f) Color code for local density of detected molecules and Nyquist limited resolution of SMLM image in (c). The freeze substitution for this cell was performed with 0.01% TA.

We applied our preparation protocol to cells expressing mGFP, mVenus or mRuby2 fusion proteins and achieved a structural resolution of ~50 nm (Fig. 3, Fig. 4 and Supplementary Fig. 2). Comparison with the conventional wide-field fluorescence images clearly shows the difference in the level of detail for correlating the fluorescently labeled structures with the EM ultrastructure. In-resin SMLM imaging yielded a resolution improvement of ~5×, which enables a much more accurate representation of the fluorescent signals with respect to the EM ultrastructure. The line profile of membrane structures in a HEK293T cell shown in Fig. 4b–e, g quantitatively represents the resolution obtained with the three imaging modalities. Conventional wide-field fluorescence microscopy doesn't allow visualization of the fine details of these structures (Fig. 4b, e), whereas SMLM resolves the two parallel running membranes containing EphA2-mVenus (Fig. 4c, e, g), as well as endocytosis of EphA2-mVenus from the plasma membrane into vesicles (arrow heads in Fig. 4c, g), both of which are clearly visible in the TEM image (Fig. 4a, d, e, g). The overlay (Fig. 4a, g) of the super-resolution fluorescence image (Fig. 4c) and TEM image (Fig. 4d) illustrates that by combining in-resin SMLM with TEM, identification and correlation of fine structural features becomes possible across both imaging modalities (see also Supplementary Fig. 2).

**Figure 5:**
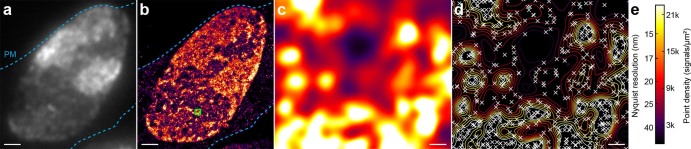
In-resin single molecule super-resolution imaging of histones. (a) Conventional wide-field fluorescence image of H2B-GFP in a HEK293T cell. Blue dashed lines indicate the plasma membrane (PM). (b) Corresponding SMLM image showing a very detailed distribution of H2B-GFP molecules in the nucleus of the cell. (c) Magnified image of region marked with green rectangle in (b). (d) Distribution of detected single molecules corresponding to image (c). Their positions are marked by crosses in a contour map of the local molecule densities. Average single molecule localization accuracy was ~15 nm, local densities of detected molecules reach up to ~40,000 per μm^2^ (~2,000 molecule positions per diffraction limited volume). Scale bars in (a) and (b) are 1 μm, in (c) and (d) 20 nm. (e) Color code for local molecule densities and corresponding Nyquist limited resolution.

Besides enhanced structural resolution, another strength of SMLM is the possibility of implementing single molecule position based analyses^44^,^45^. An example illustrating the vast amount of data on single molecule positions detected from GFP labeled histone H2B (H2B-GFP) molecules in the nucleus of a resin embedded cell is given in Fig. 5. The color code (Fig. 5e) illustrates the high degree of additional information about fluorescent molecule distribution available through in-resin SMLM imaging. Note that a density of 22,000 molecules/μm^2^ corresponds to more than 1,000 molecules per diffraction limited area. The structural resolution achieved for in-resin SMLM imaging of H2B-GFP was ~50 nm and was limited mainly by the average localization accuracy of ~15 nm. This also illustrates that our method for correlative in-resin super-resolution and electron microscopy can successfully be applied to various different target proteins.

## Discussion

Fast freezing (vitrification) of cells and tissue preserves the ultrastructure as close as possible to the native state. Maintaining the fluorescence of standard FPs throughout the process of freeze substitution allows this sample preparation procedure to be extended for correlative fluorescence and EM in resin embedded cells^19^. However, to fully exploit the strengths of fluorescence microscopy in a correlative approach, the resolution must be improved far beyond the diffraction limit of visible light. In this report we have demonstrated that the photo-switching capabilities of standard FPs can be preserved throughout high pressure freezing, freeze substitution and resin embedding, and can be utilized for single molecule localization based super-resolution imaging. Other fast freezing techniques, such as plunge freezing, should not have an effect on the photo-switching properties of FPs in resin as this part of the sample preparation procedure does not alter the fluorophores^15^,^16^. The use of tannic acid during freeze substitution was vital for successful in-resin SMLM data acquisition. Tannic acid is widely used as a mordant for osmium tetroxide^37^,^46^, but it also interacts with other heavy metals, including uranyl acetate^47^. Since both uranyl acetate and tannic acid are known to protect against extraction and the structural changes induced by dehydration and embedding^46^, one possibility is that uranyl acetate and tannic acid complexes bind to FPs, thereby creating highly stable ternary structures that are resistant to the scavenging effects of acetone, which results in overall improved fluorescence preservation and more intact fluorescent molecules available for SMLM imaging. Furthermore, tannic acid may stabilize the pH at a value that is favorable for the photo-switching. On the other hand, higher concentrations of tannic acid in the freeze substitution medium can have negative effects on the preservation of ultrastructure^39^ and we did observe this to a small degree with 0.1% tannic acid in the present study. However, whilst the ultrastructure was well preserved and contrasted with 0.01% tannic acid, the in-resin photo-switching of the FPs was less consistent compared to samples freeze substituted with 0.1% tannic acid. Thus, for correlative in-resin super-resolution imaging it is important to identify a suitable concentration of tannic acid for the best result with a particular biological application. Optimized conditions for in-resin photo-switching of standard FPs allowed us to achieve a structural resolution in the range of 40–50 nm in mammalian cells. Compared to conventional CLEM this corresponds to an approximately 25-fold increase in information content regarding the distribution of fluorescent molecules that can be used directly for the correlation with EM data. Thus, SMLM with FPs in resin embedded cells also offers a powerful alternative to immunogold labeling, which can be relatively low in detection efficiency and/or specificity^48^,^49^.

The use of standard FPs allows for a wide range of biological applications. We achieved a structural resolution of ~50 nm with data acquisition times of less than 5 min, similar to what has been reported previously for SMLM using standard FPs in un-embedded cells^32^,^43^. Special photo-activatable or photo-switchable FPs with improved photon yield, or organic dyes coupled to target proteins via SNAP- or Halo-tags^50^, could increase the resolution even further, but make the biological system less versatile and might require data acquisition schemes with several hours of imaging^9^. Better preservation of fluorescence can also be achieved through chemical reactivation of the FPs embedded in resin by addition of alkaline buffer during imaging^51^, but the effect on FP photo-switching capabilities has not yet been determined.

The method presented here also facilitates super-resolution CLEM of tissues and whole organisms (e.g. *C. elegans*), and opens the door for several advanced in-resin CLEM applications with standard FPs. It enables multi-color super-resolution CLEM to be performed, for correlating different fusion proteins to the EM ultrastructure in the same cell at a resolution far beyond the diffraction limit of light. Furthermore, it can also be applied to the burgeoning field of three dimensional CLEM^35^, for instance through combining SMLM imaging with array tomography on serial sections.

Besides CLEM, this method also provides a powerful alternative to optical sectioning for super-resolution imaging deep in the sample^52^. For example, the study of nuclear architecture with super-resolution microscopy techniques is substantially improved if background fluorescence is removed. Additionally, physical sections provide better optical conditions compared to optical sectioning, as the detected fluorescent light is not affected by aberrations which are inevitable in thick samples^53^.

In summary, we have demonstrated that the photo-switching capabilities of standard FPs, such as mGFP, mVenus and mRuby2, can be preserved in resin embedded cells after high pressure freezing and freeze substitution at a level that permits in-resin SMLM with a structural resolution of ~50 nm. This enables true correlative super-resolution fluorescence and electron microscopy imaging (in contrast to protein localization alone) of cellular structures in the absence of chemical fixation. Sub-diffraction-limit fluorescence imaging with superior structural preservation addresses the two most important issues of CLEM. The method presented here therefore introduces a powerful new CLEM technique for studying biological nano-structures in a correlative manner that does not require the use of specialized fluorophores and can be performed with any standard SMLM setup.

## Methods

### Vectors and transfection of HEK293T cells

EphA2 (V255D + L254D + I2570D) was cloned into the AgeI-KpnI sites of modified pHLSec vectors^54^ coding also for C-terminal mVenus^41^ or mRuby2^55^, followed by a hexa-histidine tag. The H2B-GFP plasmid^56^ was kindly provided by the L. Schermelleh lab (Oxford). The EphA4-mGFP construct^57^ was kindly provided by the R. Klein lab (Martinsried). Adherent HEK293T cells were grown in DMEM supplemented with 10% fetal bovine serum (Gibco), 1× glutamine (Gibco) and 1× non-essential amino acids (Gibco). Cells were transfected with Fugene 6 transfection reagent (Promega) according to the manufacturer's instructions and processed after 20–28 h.

### High pressure freezing

Transfection efficiency (typically > 50%) and fluorescence level were verified immediately prior to cryofixation using an inverted epifluorescence microscope (Axiovert 200, Zeiss). HEK293T cells were dislodged from the culture plate by gentle tapping and pipetting. The resulting cell suspension (~5 ml) was spun in a benchtop centrifuge at 500 rpm for 1 min. The supernatant was removed and cells were resuspended in 100 μl cryoprotectant (20% BSA in 0.1 M PIPES pH 7.2) pre-warmed to 37°C, then pelleted for 30 s at 10,000 rpm. Alternatively, HEK293T cells were trypsinized and pelleted at 1000 rpm for 2 min in pre-warmed 0.1 M PIPES pH 7.2 containing 20% BSA and 5% fetal bovine serum, which resulted in less clumping of the cells and better resin infiltration. The supernatant was removed and ~1 μl of the resulting cell slurry was loaded into the membrane carrier (Leica), which was then immediately vitrified using a high pressure freezer (EMPACT2, Leica).

### Freeze substitution, resin embedding and sectioning

A variety of freeze substitution protocols and cocktails were tested to optimize fluorescence, photo-switching and contrast for TEM. Frozen carriers were transferred under liquid nitrogen to freeze substitution media in an automated freeze substitution unit (AFS2, Leica) held at −130°C. Freeze substitution was carried out either in 1 ml of freeze substitution medium in cryo-vials or 5 ml of freeze substitution medium in Leica specimen holders. The freeze substitution media were made fresh for each experiment and consisted of 0.2% uranyl acetate (diluted from a 5% stock in methanol), 0–0.1% low molecular weight tannic acid (Electron Microscopy Sciences; working solution was diluted from a 10% stock in acetone) and 5% water in pure acetone. The combination of tannic acid and uranyl acetate produced a dark reaction product. Platinum blue and Lanthanum stains at 1:20 dilutions were tested as substitutes for uranyl acetate, but did not provide sufficient contrast in cells for TEM imaging. The freeze substitution protocol was adapted from Hawes et al.^40^ as follows: −130°C to −90°C at a rate of 20°C/h, held at −90°C for 6 h, warmed to −45°C at a rate of 5 C/h then held at −45°C 1–2 h prior to washing and resin infiltration at −45°C.

Samples were washed three times with pure acetone over a period of 60–90 min, transferred to Leica specimen holders if required, and infiltrated for 2–3 h each with 25/50/75% dilutions of Lowicryl HM20 monostep resin (PolySciences). Samples were then incubated overnight in 100% resin, followed by a further three changes of resin the next day over an 8 h period before polymerizing with 360 nm UV light for a total of 48 h. The first 12 h of polymerization were done at −45°C, during which the samples were covered with foil and indirectly exposed to UV light. The cover was then removed and samples were warmed to 0°C at 5°C/h and polymerized for a further 8 h, then left in the fumehood at room temperature for 1–2 days. However, resin polymerization was often variable, since acetone can act as a radical scavenger and impede Lowicryl polymerization^20^. We found that after one wash step with acetone, using pure ethanol instead for the remainder of the washing and infiltration procedure resulted in better quality sections with less tears/holes. The use of ethanol did not affect the in-resin fluorescence or photo-switching.

Blocks were cut away from the mold and the carriers removed by submerging the very tip of the block into liquid nitrogen, then scraping away the resin over the top of the carrier and snapping them off with tweezers to expose the cell pellet. Blocks were sectioned using a 45° diamond knife (Diatome) on an ultramicrotome (Ultracut7, Leica). 100–150 nm sections were collected onto 0.6% formvar coated 200 mesh Cu alpha numeric finder grids (Electron Microscopy Sciences) and imaged using SMLM the same day. Fluorescence quality in cut sections reduced over time, but fluorescence in the blocks stored in the dark at room temperature was stable over many months, as previously reported^19^, so that blocks could be re-sectioned and imaged as required.

### SMLM imaging

Grids were mounted between a slide and glass coverslip (0.17 μm thickness) with 20 μl of a glycerol based antifade mounting medium (AF4, Citifluor) and sealed with nail polish. SMLM imaging was performed using a 100× 1.4 NA oil objective (UPlanSApo, Olympus) on an OMX (optical microscope experimental V2, API) setup. The microscope was modified (addition of a second wide-field excitation path to allow an adjustable excitation volume to increase accessible laser intensity if necessary; for details see Supplementary Fig. 3) to enable SMLM with conventional FPs according to Lemmer et al.^32^. For SMLM imaging of mGFP, mVenus and mRuby2 the intensity of a 488 nm laser (for green and yellow FPs) or a 593 nm laser (for red FPs) was adjusted to 2–10 kW/cm^2^ in the object plane, driving the fluorescent molecules into a long-lived dark state. Stochastic recovery to the fluorescent state was recorded with an EMCCD camera (Evolve Delta, Photometrics) with an integration time of 50 ms and a frame rate of 14–17 fps. We used a maximum-likelihood-based algorithm with a sliding window for background subtraction^42^, which was adapted to the hardware configuration of the microscope setup, for the position determination of the single fluorescent molecules. For samples with very high fluorophore densities an increased background could be observed, due to molecules not undergoing photo-switching, which has an effect on the achievable single molecule localization accuracy. However, this population photo-bleaches (irreversibly or is transferred into a very long-lived dark state) during the SMLM measurement. For the example of H2B-GFP expressed in HEK293T cells (Fig. 5) we observed an improving average single molecule localization accuracy over the course of the SMLM measurement (15,000 frames recorded at 17 fps after several seconds of pre-bleaching) from ~17 nm at the beginning to ~13 nm at the end. The values have been determined based on nearest neighbor distances in adjacent frames, a method for measuring the single molecule localization accuracy independent of estimations based on photon numbers^58^. Super-resolution images were generated from the SMLM position data based on nearest neighbor distances to also consider the Nyquist limited resolution^43^,^59^. The stochastic scattering of the detected molecule positions does not allow for a direct translation of the mean nearest neighbor distance to the mean (local) molecule density. Therefore, for every molecule position the nearest 20 neighboring positions were considered for the determination of its corresponding average nearest neighbor distance (including a normalization factor to correct for the fact that not all neighbors can be arranged in a circular symmetry). After SMLM imaging, the grids were recovered by removing the nail polish and the glass coverslip and passing the grids over 1–3 drops of water to remove the mounting medium.

### Post staining and TEM imaging

Grids were post-stained for 10 min with 2% aqueous uranyl acetate, washed by passing over five warm water droplets and then stained for a further 10 min with Reynold's lead citrate^60^ and washed as above. Samples were imaged in a transmission electron microscope (Tecnai12, FEI) operated at 120 kV using a digital CCD camera (Ultrascan US1000, Gatan).

### Correlation of fluorescence and TEM images

To create overlay images of SMLM and TEM of the fluorescently labeled cells in resin section distinct features in both images were identified and their positions marked using the *Control Point Selection Tool* of MATLAB (Mathworks). The positions of the same features in both images allowed determination of the transformation between the coordinate system of the SMLM image and the TEM image. For the biological samples presented here, structures carrying a fluorescent marker were also clearly visible in the TEM images and could directly be used for the coordinate transformation. In other cases fiducial markers might be needed for correlating fluorescence and EM images^28^. For in-resin CLEM, a liner conformal transformation is sufficient if no significant shrinkage occurs during TEM data acquisition. Otherwise an affine transformation can be applied to improve the correlation accuracy.

## Author Contributions

R.K. and E.J. designed and performed the experiments and wrote the manuscript. E.J. and E.S. prepared the samples. R.K. processed and analyzed the data. K.G., I.D., E.Y.J. and E.S. provided advice and edited the manuscript.

## Supplementary Material

Supplementary Information Supplementary Information
