# Corrigendum: Correlative in-resin super-resolution and electron microscopy using standard fluorescent proteins

**DOI:** 10.1038/srep22681

**Published:** 2016-03-08

**Authors:** Errin Johnson, Elena Seiradake, E. Yvonne Jones, Ilan Davis, Kay Grünewald, Rainer Kaufmann

Scientific Reports 5: Article number: 958310.1038/srep09583; published online: 03312015; updated: 03082016

This Article contains an error in the Methods section under subheading ‘Freeze substitution, resin embedding and sectioning’.

“The first 12 h of polymerization were done at −45 °C, during which the samples were covered with foil and indirectly exposed to UV light. The cover was then removed and samples were warmed to 0 °C at 5 °C/h and polymerized for a further 8 h, then left in the fumehood at room temperature for 1–2 days.”

should read:

“The first 12 h of polymerization were done at −45 °C, during which the top of the samples were covered with foil and indirectly exposed to UV light. The cover was then removed and samples were polymerized for a further 12 h at −45 °C, then warmed to 0 °C over a 12 h period and polymerized at 0 °C for a further 12 h. Samples were then left in the fume hood at room temperature for 1–2 days.”

