# Supplementary material for: Correlative in-resin super-resolution and electron microscopy using standard fluorescent proteins
Source: Sci Rep. 2015 Mar 31;5:9583. doi: 10.1038/srep09583 (PMC4379466; doi:10.1038/srep09583)
Supplement: Supplementary Information [file srep09583-s1.pdf]

SUPPLEMENTARY INFORMATION

Correlative in-resin super-resolution and electron microscopy using standard fluorescent proteins

Errin Johnson[1], Elena Seiradake[2], E. Yvonne Jones[2], Ilan Davis[3], Kay Grünewald[2], Rainer Kaufmann[2,3,]*

[1]Sir William Dunn School of Pathology, University of Oxford, South Parks Road, Oxford, OX1 3RE, UK

[2]Division of Structural Biology, Wellcome Trust Centre for Human Genetics, University of Oxford, Roosevelt Drive, Oxford, OX3 7BN, UK

[3]Department of Biochemistry, University of Oxford, South Parks Road, Oxford, OX1 3QU, UK

[*] to whom correspondence should be addressed: rainer@strubi.ox.ac.uk

Supplementary Figures

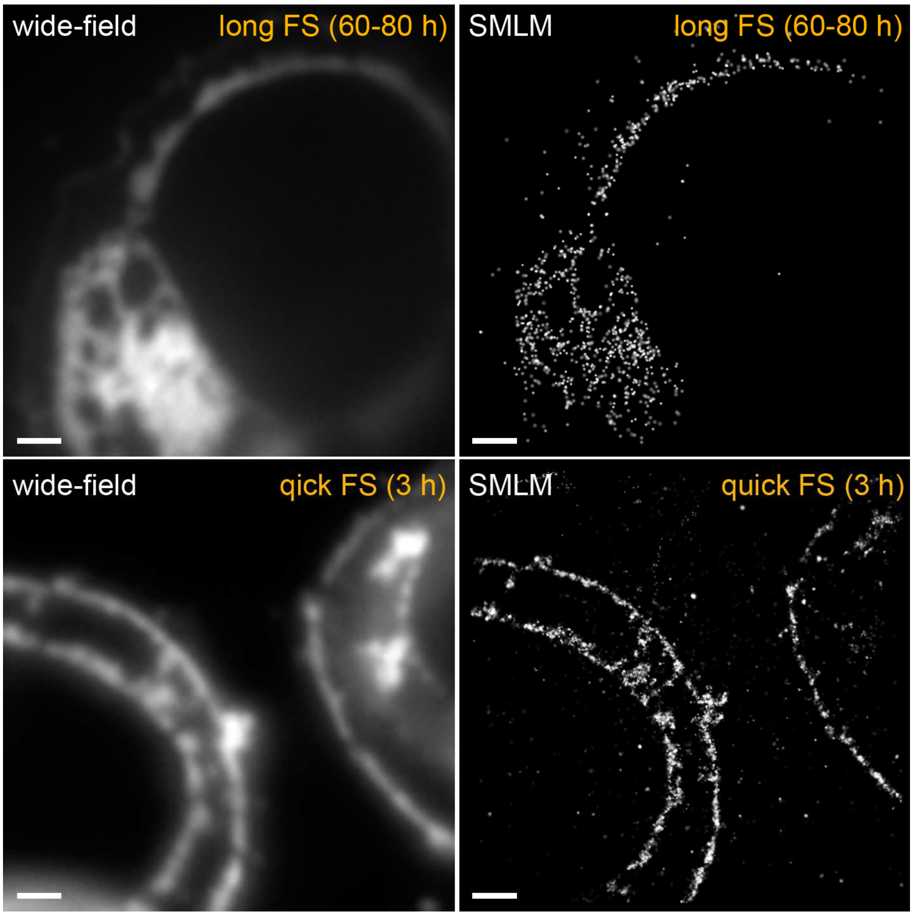

Supplementary Figure 1. Comparison of different lengths for the freeze substitution (FS). Fluorescence preservation of conventional wide-field imaging was much less affected than the photo-switching capabilities. The quick freeze substitution with a length of 3 h allowed substantially better single molecule localization than a freeze substitution of 60-80 h. Both experiments were performed with the same parameters except the length of the freeze substitution. In both cases 0.1% tannic acid was added to the freeze substitution medium. PBS was used for mounting the grids. The scale bars are 1 µm.

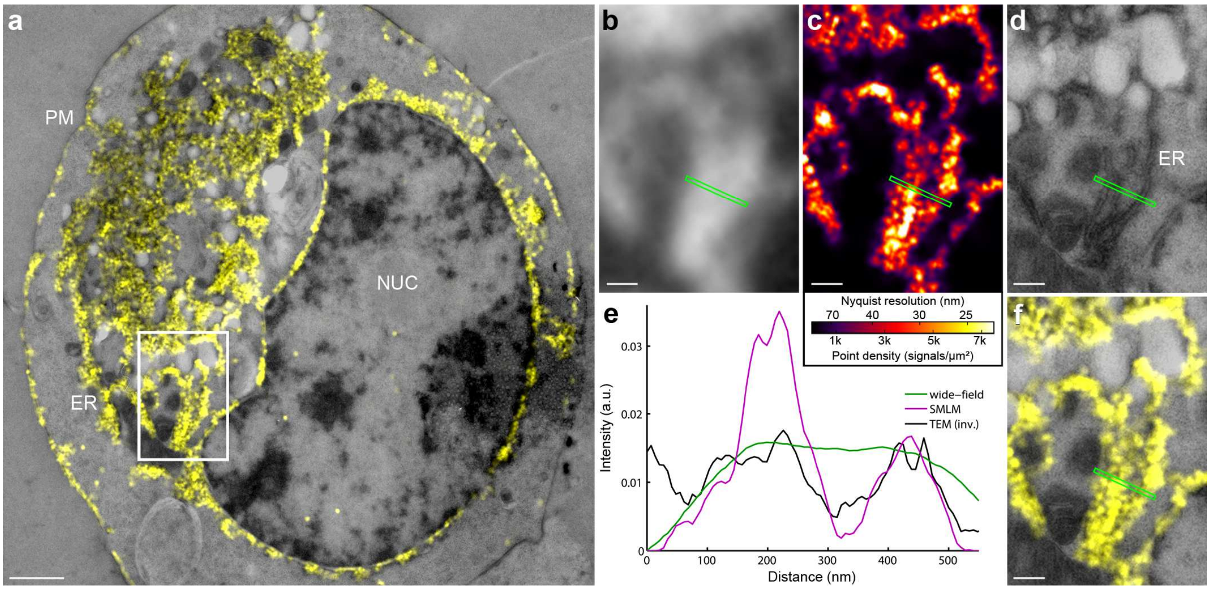

Supplementary Figure 2. Resolution comparison of in-resin fluorescence imaging. (**a**) Overlay of SMLM image on the TEM image of EphA2-mVenus in a resin embedded HEK293T cell. The rectangle marks the region shown with a higher magnification in the panels on the right: (**b**) Conventional wide-field fluorescence microscopy, (**c**) Super-resolution SMLM and (**d**) TEM. The line profiles in (**e**) show the different levels of details of ER structures resolvable with each technique. The structural resolution of ~50 nm achieved with SMLM in resin sections using standard fluorescence proteins (here mVenus) allowed a superior correlation of fluorescent signals and EM ultrastructure (**a**,**f**). Scale bar is 1 µm in (**a**) and 250 nm in (**b**,**c**,**d**,**f**). Note that the line profile for the TEM image was generated from inverted pixel values for better comparison.

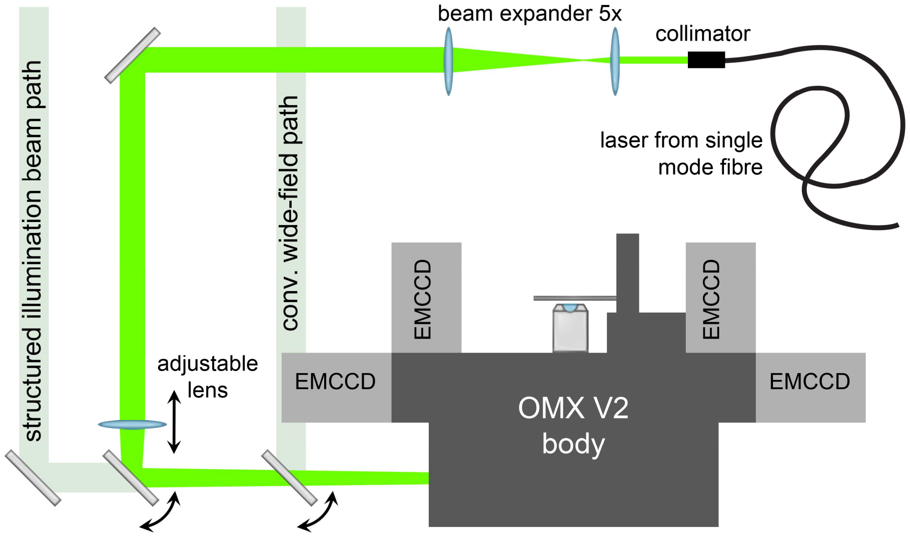

Supplementary Figure 3. Schematic drawing of the modification of the OMX V2 microscope (API) for SMLM imaging. The laser light for the additional excitation beam path is coupled into a single mode fiber (SMC-460Si, Schäfter+Kirchhoff) using a standard fiber coupler, collimated and expanded to a beam diameter of ~3.2 mm. For adjusting the illuminated area in the object plane an additional lens (focal length 500 mm) is placed before the lens in the microscope body focusing the light into the back aperture of the objective (100x, 1.4 NA, UPlanSApo, Olympus). Changing the distance between those two lenses changes the illuminated area in the object plane and thus the illumination intensity. The pre-existing beam path for structured illumination and conventional wide-field imaging are not affected and can be selected by flip mirrors in front of the microscope body.
